# Dataset of effect of Wogonin, a natural flavonoid, on the viability and activation of NF-κB and MAPKs in IL-1β-stimulated human OA chondrocytes

**DOI:** 10.1016/j.dib.2017.03.054

**Published:** 2017-04-09

**Authors:** Nazir M. Khan, Abdul Haseeb, Mohammad Y. Ansari, Pratap Devarapalli, Sara Haynie, Tariq M. Haqqi

**Affiliations:** Department of Anatomy & Neurobiology, Northeast Ohio Medical University, 4209 St Rt 44, Rootstown, OH 44272, USA

**Keywords:** Osteoarthritis, Nrf2, Wogonin, NF-κB, MAPK, Mass-spectrometry

## Abstract

This article contains data related to the article “Wogonin, a plant derived small molecule exerts potent anti-inflammatory and chondroprotective effects through activation of ROS/ERK/Nrf2 signaling pathways in human Osteoarthritis chondrocytes” (Khan et al. 2017) [1]. The data are related to effects of Wogonin on the viability and IL-1β-stimulated activation of NF-κB and ERK1/2, JNK1/2 and p38 MAPKs in human OA chondrocytes. Gene expression data representing the chondrogenic phenotype and the efficiency of Nrf2 knockdown in monolayer culture of human OA chondrocytes were shown. Moreover, mass spectrometric calibration curve of Wogonin used to quantify the intracellular uptake were also presented. The data are presented in the form of figures and significance of these has been given in the research article (Khan et al. 2017) [1].

**Specifications Table**

**Table:** 

Subject area	*Biology*
More specific subject area	*Osteoarthritis, Cell Signaling, Inflammation*
Type of data	*Figures*
How data was acquired	*For the analysis of protein expression, Western blot analysis using specific antibodies were used*[2].
	*For the analysis of mRNA expression, Sybrgreen and Taqman assay were performed*[2].
	*For the generation of four point calibration curve LC–MS/MS analysis of Wogonin was performed using UHPLC system connected to a triple quadrupole mass spectrometer (LC–MS 8040; Shimadzu, Kyoto, Japan).*
	*For viability analysis MTT assay and PI staining were used*[2].
Data format	*Analyzed*
Experimental factors	*Human OA chondrocytes were pretreated with Wogonin for* 2 h *at* 37 °C *and then stimulated with IL-1β.*
Experimental features	*qPCR, Western blot, Transfection, Flowcytometry, LC–MS/MS*
Data source location	*Northeast Ohio Medical University, Rootstown, Ohio, USA*

We confirm that data provided in the article are original and have not been published elsewhere.

**Value of the data**

1. The datasets provide evidence in the favor of chondrogenic phenotype of OA chondrocytes in monolayer culture.
2. The data of cell cycle analysis using propidium iodide staining provides proof for the non-proliferative nature of monolayer culture of human OA chondrocytes.
3. The datasets provide the efficiency of nucleofection in human OA chondrocytes to deplete the expression of gene of interest. This data set may assist researchers to choose the appropriate method of transfection in chondrocytes, the cell type difficult to transfect.
4. These data will be of value to the scientific community working in the area of osteoarthritis and nutraceuticals since they illustrate the therapeutic targets of OA beyond NF-κB and MAPKs.
5. The data about mass-spectrometric calibration curve of Wogonin are promising and may be valuable for the uptake studies of flavonoids in the cells.

## 1 Data

**Fig. 1:**
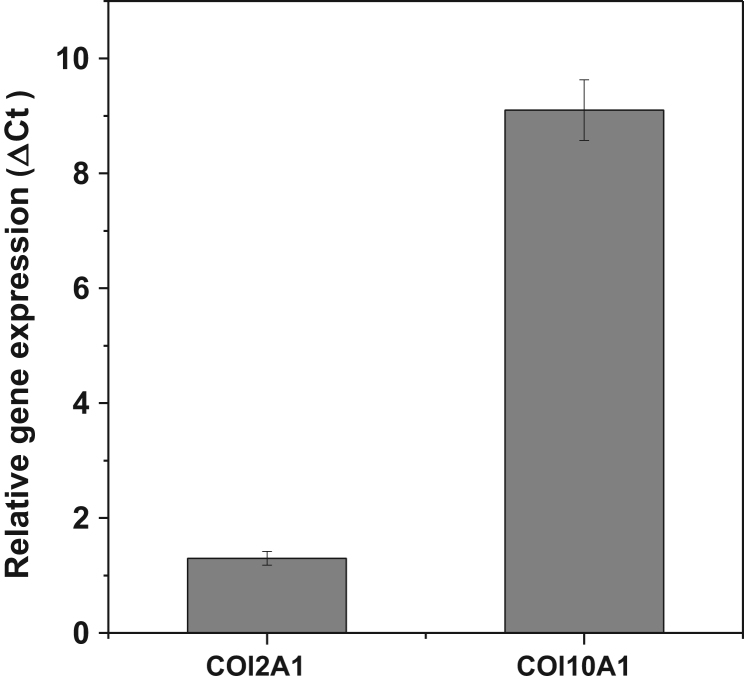
Expression of COL2A1 and COL10A1 in monolayer culture of human OA chondrocytes: Human OA chondrocytes were cultured in monolayer and total RNA were isolated and mRNA expression of COL2A1, and COL10A1 were quantified using TaqMan assay. Gene expression was determined by the ΔCt method and results were normalized to β-actin expression.

**Fig. 2:**
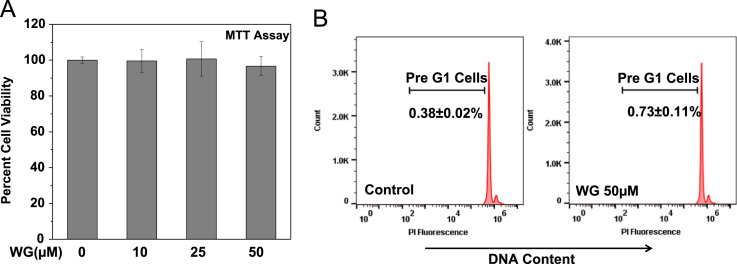
Effect of Wogonin (WG) on viability of human OA chondrocytes. (A) Human OA chondrocytes (0.02×10^6^/ well of 96-well plate) were treated with Wogonin (10–50 µM) for 24 h and cell viability was measured by MTT assays. Chondrocytes treated with 0.1% DMSO served as control. Viability was expressed relative to control cells. (B) Flow cytometric histograms of OA chondrocytes treated in the presence or absence of Wogonin and stained with PI. Cells were harvested at 24 h after treatment with Wogonin and stained with PI. Twenty thousand cells in each group were acquired using a flowcytometer. The Sub-G1 region represents the percentage of cells undergoing apoptosis. Data points represent mean±SD from two subjects.

**Fig. 3:**
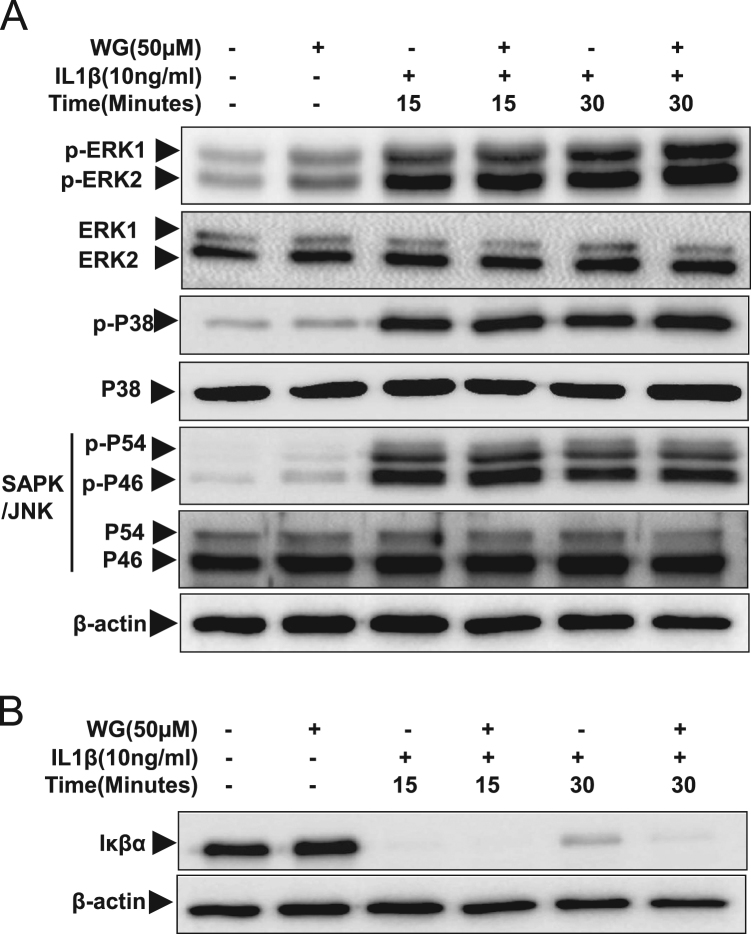
Effect of Wogonin (WG) on IL-1 β induced activation of MAPKs and NF-κB in OA chondrocytes. Human OA chondrocytes were pre-treated with Wogonin (50 µM) for 2 h followed by treatment with IL-1β (10 ng/ml) for 15 or 30 min. Cell lysate were prepared from harvested chondrocytes using RIPA lysis buffer. (A) Activation of MAPKs was investigated by immunoblotting using primary antibodies specific for phospho-ERK1/2, phospho-JNK, phospho-p38. Expression of total ERK1/2, total JNK, or total-p38 was used as control. (B) Degradation of Iκβα was investigated by immunoblotting in cell lysate prepared as above. β-actin was used as a control for equal loading. Immunoblot are representatives of two blots performed on samples obtained from two individuals.

**Fig. 4:**
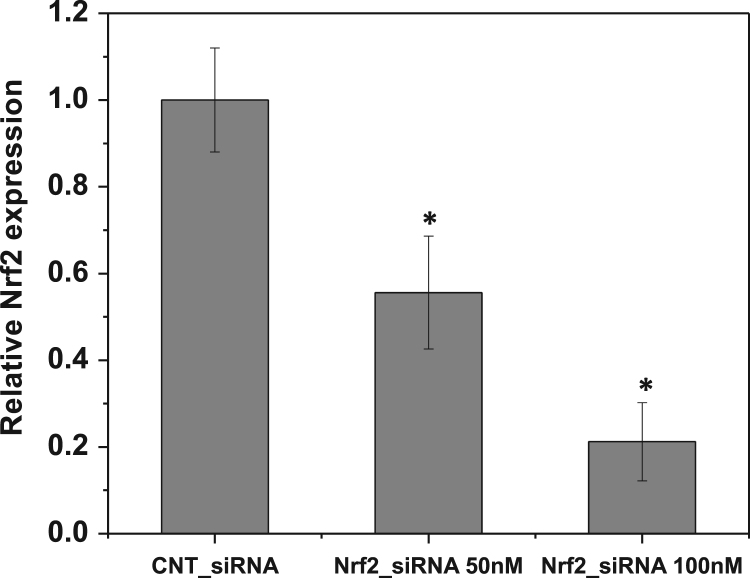
Genetic ablation of Nrf2 using specific siRNA in chondrocytes: OA chondrocytes were transfected with siRNA specific for Nrf2 (50, 100 nM) using nucleofection. Forty-eight hours after the transfections, RNA was isolated form OA chondrocytes and gene expression of Nrf2 was measured by quantitative PCR using the SYBR® green assay (Life Technologies). Bar graph represents mean±SD from two subjects. *p≤0.05, as compared to control siRNA.

**Fig. 5:**
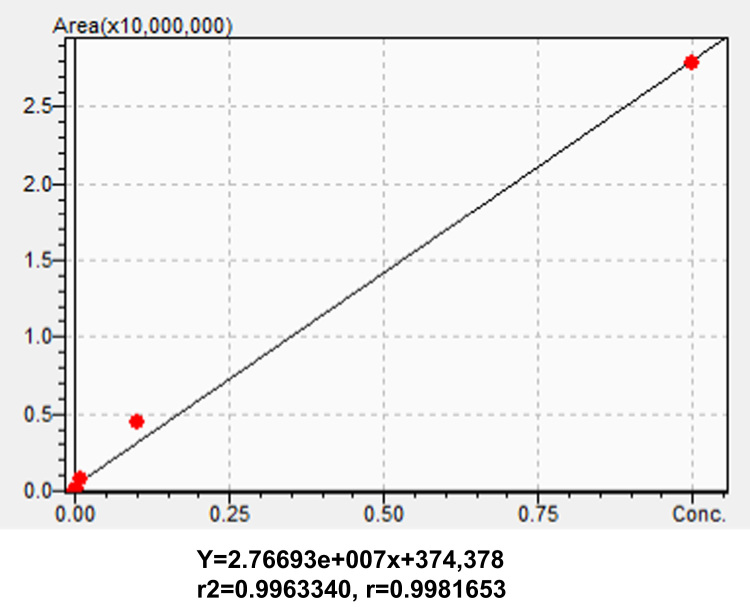
Mass-spectrometric calibration curve of Wogonin: Four point calibration curve was prepared using purified Wogonin dissolved in methanol at the concentrations of 0.001, 0.01, 0.1 and 1 mg/ml using multiple reaction monitoring and negative ion mode (MRM-) by monitoring transition pairs *m*/*z* 283.00 (precursor ion)/162.9 (product ion).

These data were related to the article “Wogonin, a plant derived small molecule exerts potent anti-inflammatory and chondroprotective effects through activation of ROS/ERK/Nrf2 signaling pathways in human Osteoarthritis chondrocytes” (Khan et al. 2017) [1]. The dataset presents here, illustrate the chondrogenic phonotype of cultured OA chondrocytes (Fig. 1) and represents the effects of natural flavonoid Wogonin on the viability (Fig. 2A and B), and IL-1β-stimulated activation of NF-κB and ERK1/2, JNK1/2 and p38 MAPKs in human OA chondrocytes (Fig. 3A and B). Fig. 4 shows the evidence of effective depletion of target gene expression using specific siRNA in chondrocytes. Fig. 5 presents four point calibration curve generated to quantify the intracellular concentration of Wogonin in chondrocytes.

## 2 Experimental design, materials and methods

### 2.1 Total RNA isolation and real time PCR

Total RNA from chondrocytes were isolated using Trizol® reagent and cDNA was synthesized from 1 µg of total RNA using high-capacity cDNA reverse transcription kit (Life Technologies) [2]. The mRNA expression of COL2A1 and ACAN was quantified using TaqMan Gene Expression Assays and relative expression levels were calculated using the 2−ΔΔC_T_ method [2].

### 2.2 Viability assay

Primary human OA chondrocytes were serum starved overnight, and then treated with Wogonin (10–50 µM) for 24 h in serum free medium and viability was determined using MTT assay and cell cycle analysis using propidium iodide staining [2]. A total of 20,000 cells were acquired in BDAccuri C6 Flowcytometer and percent apoptotic cells were determined by analyzing a sub-G1 population (<2*n* DNA content) using FlowJo software.

### 2.3 Western immunoblotting

Chondrocytes (1×10^6^/well of 6-well plate) were pretreated with Wogonin (50 µM) for 2 h followed by treatment with IL-1β (10 ng/ml) for 15–30 min. After treatments, OA chondrocytes were harvested, washed with cold PBS and lysed in ice-cold RIPA buffer and lysate were prepared [2]. Equivalent amounts of lysate protein (20 µg) were resolved by 10% SDS-PAGE and transferred to a PVDF membrane (Bio-Rad, USA) and the blots were incubated with primary antibodies diluted in 2% blocking buffer for overnight at 4 °C. Blots were then incubated with horse radish peroxidase-conjugated secondary antibody, followed by washing with TBST. Blot were developed using Luminata Western HRP substrate (EMS Millipore) and the antibody reactive proteins were visualized by chemiluminescence and imaged using the Pxigel imaging system (Syngene, Frederick, MD).

### 2.4 siRNA mediated depletion of Nrf2 expression using nucleofection

Primary human OA chondrocytes were transfected with 50–100 nM Nrf2 siRNA (SMARTpool: ON-TARGETplus NFE2L2 siRNA Dharmacon, Lafayette, CO, USA) or MISSION^®^ siRNA Universal Negative Controls (Sigma Aldrich, St. Louis, MO) using P3 Primary Cell 4D-Nucleofector^™^ X Kit on 4D-Nucleofector equipment (Lonza, Walkersville, MD) following the instructions provided by the manufacturers. Transfected cells were plated in 6-well plates. Forty-eight hours after the transfection RNA were isolated and cDNA was synthesized. Gene expression levels of Nrf2 was measured by quantitative PCR using the Sybrgreen assay system.

### 2.5 Calibration curve of Wogonin

The calibration curve of Wogonin was generated using LC–MS/MS analysis using a UHPLC system connected to a triple quadrupole mass spectrometer (LC–MS 8040; Shimadzu, Kyoto, Japan) equipped with an electrospray ionization source. The quantitation was performed using the ESI ion source in multiple reaction monitoring and negative ion mode (MRM-) by monitoring transition pairs *m*/*z* 283.00 (precursor ion)/162.9 (product ion). The following instrument settings were used for MRM analysis: heat block temperature, 400 °C; DL temperature, 250 °C; nebulizing gas (N_2_), 3 L/min; drying gas (N_2_), 15 L/min; collision energy, 35.0; dwell time, 100 ms. Four point calibration curve was prepared using pure Wogonin dissolved in methanol at the concentrations of 0.001, 0.01, 0.1 and 1 mg/ml.
